# EEG Dynamics in Children Before, During and After General Anesthesia

**DOI:** 10.1002/pan.70156

**Published:** 2026-03-05

**Authors:** Maximilian Markus, Feidias Panagiotou, Claudia Spies, Susanne Koch

**Affiliations:** ^1^ Department of Anaesthesiology and Intensive Care Medicine (CCM, CVK) Charité—Universitätsmedizin Berlin, Corporate Member of Freie Universität Berlin and Humboldt‐Universität zu Berlin Berlin Germany

**Keywords:** EEG, neuromonitoring, pediatric anesthesia

## Abstract

**Background:**

Age‐specific EEG signatures during anesthesia are described in pediatrics, and perioperative monitoring is increasingly advocated; yet most indices and algorithms derive from adult data and may not generalize to early development.

**Aims:**

The purpose of this study was to characterize perioperative frontal EEGs in young children younger than 8 years.

**Methods:**

A total of 147 frontal EEGs from children ranging from 1 month to 8 years of age were recorded prospectively under general anesthesia at Charité—Campus Virchow Klinik (CVK). For data acquisition, the Narcotrend Monitor was used, and the raw EEG files were further analyzed in their frequency bands. The patient cohort was divided into four age groups (0–5, 6–11, 12–23, and > 24 months), and EEG signatures were compared between the age groups.

**Results:**

Delta activity is the predominant frequency in all age groups already in the awake state before induction of anesthesia, with a step increase at loss of consciousness, which is more pronounced in older children. Intraoperatively, alpha‐ and beta‐activity emerge at the age of 6 months and are greater in the older age groups. Infants (0–5 months) remain with a high amount of Delta activity intraoperatively. With the return of consciousness, the faster frequencies gradually decrease, and the EEG is characterized again by a predominant delta‐activity in all age groups.

**Conclusions:**

In this study, we characterized differences in the perioperative EEG signatures of children from 1 month to 8 years from the preoperative awake state during induction and general anesthesia until they regained consciousness from general anesthesia. The EEG readouts differ across age groups, and age‐adapted monitoring systems are needed to protect this vulnerable patient group from over‐ and undersedation.

**Trial Registration:**

This study was approved by the Charité—University Medicine Berlin's ethics committee (EA2/027/15) and was registered at clinicaltrials.gov (23rd of June 2015/NCT02481999)

## 1 Introduction

General anesthesia is administered to children for surgical procedures every day across the globe. Although it is essential for surgery, concerns regarding its potential impact on the developing brain have been growing. Although these effects are not immediately observable, evidence from preclinical studies and emerging clinical data suggests that general anesthetics may have long‐term consequences for the central nervous system [1, 2].

An emerging approach to optimize anesthetic care and possibly mitigate the risk of anesthetic oversedation is the electroencephalographic‐based neuromonitoring, which provides real‐time information about brain activity and anesthesia depth. EEG monitoring during anesthesia has been extensively characterized in adults, where GABAergic anesthetics produce stereotyped oscillatory patterns including slow‐delta waves, frontal alpha‐oscillations, and anteriorization of alpha‐power from posterior to frontal regions [3]. However, Pediatric Anesthesia presents fundamentally different neurophysiological challenges due to ongoing brain development and maturation of GABAergic circuits.

Pioneering work by Cornelissen et al. demonstrated that EEG patterns during sevoflurane anesthesia in infants aged 0–6 months differ markedly from adult patterns. Their multichannel EEG studies revealed that while slow‐delta oscillations are present across all ages, theta and alpha oscillations emerge only around 4 months of age, and crucially, infants lack the frontal alpha predominance and coherence characteristic of anesthetized adults. These developmental differences likely reflect ongoing synaptogenesis, regional variations in glucose metabolism, and progressive myelination across the cortex [4].

Studies examining sevoflurane across pediatric age groups have similarly demonstrated progressive increases in alpha power with age, suggesting that alpha oscillations may serve as useful markers of anesthetic depth in older children but not in infants [5].

Despite this growing literature, several limitations remain. Most studies have focused on narrow age ranges, and few have systematically characterized the complete perioperative trajectory from awake baseline through loss of consciousness, intraoperative maintenance, and emergence. Additionally, current depth‐of‐anesthesia monitors remain primarily derived from adult algorithms and may not accurately reflect anesthetic states in young children, as demonstrated by studies showing that BIS values fail to correlate with clinical endpoints in pediatric populations [6].

### 1.1 Aim of the Study

The aim of this study was to characterize EEG signals in children during the perioperative period—from the preanesthetic awake state, induction of anesthesia, and intraoperative general anesthesia through emergence from general anesthesia—to improve our understanding of pediatric brain responses under general anesthesia and to develop future EEG neuromonitoring algorithms.

## 2 Methods

### 2.1 Data Collection and Participant Demographics

In this prospective clinical observational study, electroencephalogram (EEG) data were recorded from children (0.5–8 years) with approval from the Charité—University Medicine Berlin's ethics committee (EA2/027/15). The study was registered at clinicaltrials.gov under the number NCT02481999. The data were collected at the Campus Virchow Klinik (CVK) of the Charité—Universitätsmedizin Berlin, which spans from 08.09.2015 to 24.05.2017.

EEG data from infants (0–0.5 years) (ClinicalTrials.gov: NCT04093661) were drawn from the “Retrobabies” study, which retrospectively collected EEGs from infants treated at Charité between 2017 and 2019 under ethics approval EA2/115/19. The inclusion and exclusion criteria, except age, were the same as those of the prospective cohort. Patients with complete perioperative markers were identified, and the two datasets were subsequently harmonized and combined for EEG processing and final analysis.

Inclusion criteria:

- Male or female children aged 0.5–8 years.
- Planned elective surgery.
- Informed consent by both parents if both parents have joint custody.
- No planned operation in the next three months.
- No operation in the last half year before study inclusion.

Exclusion criteria:

- Known neurological or psychiatric precondition (disease).
- Inability of the parents to speak and/or read German.
- Indications for the isolation of patients with multiresistant bacteria.
- Lack of willingness to save and hand out pseudonymized data within the clinical study.
- Contact allergy to silver or silver chloride.
- Participation in another prospective interventional clinical study.

**FIGURE 1:**
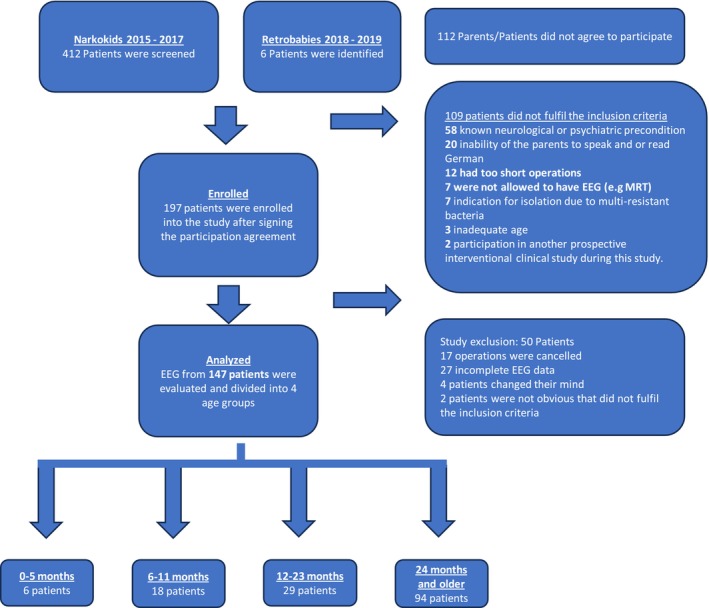
Inclusion and exclusion diagram with 412 patients screened and 147 patients included in the final analysis.

As shown in Figure 1, a total of 412 patients were screened during the study. A total of 112 parents refused to participate in the study. Furthermore, 109 patients did not fulfill the inclusion criteria, of whom 58 were diagnosed with neurological or psychiatric preconditions, 20 parents were unable to communicate sufficiently in German, 12 were planning for very short operations, seven were not allowed to have intraoperative EEG monitoring, seven were isolated due to multidrug‐resistant bacteria, three exceeded the age limit, and two patients were already participating in a different prospective study. Therefore, 197 patients were enrolled in the study. Fifty patients were further excluded because of canceled operations (*n* = 17) or incomplete EEG data (*n* = 27), parents of four children changed their minds, and two patients were later shown that they did not fulfill the inclusion criteria. The EEG data of 147 patients were analyzed, and these patients were divided into four age groups: 0–5 months (*n* = 6), 6–11 months (*n* = 18), 12–23 months (*n* = 29), and ≥ 24 months (*n* = 94).

In line with our institution's standard operating procedures, frontal EEG recordings during surgery were performed via Narcotrend Monitor (MT Monitor Technik GmbH & Co. KG, Bad Bramstedt, Germany; Software version 4.0). To ensure low impedance levels, the skin was prepared with alcohol prior to electrode placement. EEG electrodes (Ambu BlueSensor N, Bad Nauheim, Germany) were positioned at the Fz, F7, and F8 locations, with Fp2 used as the reference point. The impedance at each electrode site was maintained below 5 kΩ, with the disparity between any two electrodes remaining under 3.5 kΩ.

Recordings were captured at a sampling rate of 128 Hz, employing a bandpass filter that restricted the frequency range to 0.5–45 Hz. Using EEG‐Viewer software (MT Monitor Technik, Bad Bramstedt, Germany, Version 6.1), a 2‐min segment devoid of artifacts was selected by manual inspection for each time point.

All subsequent signal processing, including epoch segmentation, spectral computation, and band‐power extraction, was performed automatically by the integrated Narcotrend software. Specifically, contiguous 2‐min epochs were selected for analysis and subjected to a Fast Fourier Transform (FFT) to compute the power spectral density (PSD) over the full 0.5–45 Hz range. From the PSD, total power was calculated as the area under the curve across the 0.5–45 Hz band. Mean absolute band powers were then computed for the delta (0.5–3.5 Hz), theta (3.5–7.5 Hz), alpha (7.5–12.5 Hz), and beta (12.5–30 Hz) ranges. Relative band powers were derived by expressing each band's absolute power as a percentage of total power. All analyses were conducted within the Narcotrend environment, ensuring consistency in preprocessing and FFT parameterization, defined by the manufacturer's default settings.

The continuous EEGs were sub segmented into 2‐min intervals at four time points: *Baseline*: This interval was shortly before the induction of anesthesia. During this time interval, the children were awake. *Loss of consciousness (LOC)*: At this time, together with the attending anesthetist, the child no longer reacted, and the airway was secured afterwards. *Intraop*: The interval was at least 15 min after surgical incision. During this period, anesthesia was maintained, and a stable course was observed. *Return of consciousness (ROC)*: At this time, the child opened his or her eyes, or movement was observed.

### 2.2 Statistical Analysis

EEG power spectral data were analyzed using a comprehensive multi‐approach statistical framework designed to address both cross‐sectional age‐related differences and longitudinal perioperative changes. Raw power spectral density values in μV^2^ were log‐transformed to decibels (dB) using the formula Power (dB) = 10 × log_10_ (Power [μV^2^]) to normalize distributions.

#### 2.2.1 Cross‐Sectional Age Group Comparisons

Two complementary analytical approaches were employed to compare age groups at each perioperative timepoint:

##### 2.2.1.1 Non‐Parametric Analysis

Kruskal‐Wallis H‐tests were performed to compare EEG power across four age groups (0–5, 6–11, 12–23, and ≥ 24 months) at each timepoint (baseline, LOC OP, and ROC) for each frequency band (total power, beta, alpha, theta, and delta). Given the non‐normal distribution of EEG power data and unequal sample sizes across age groups, this non‐parametric approach provided robust omnibus testing. Significant Kruskal‐Wallis tests were followed by post hoc pairwise Mann–Whitney *U* tests with Bonferroni correction for multiple comparisons (*α* = 0.05/6 = 0.0083 for six pairwise comparisons). Effect sizes were quantified using eta‐squared (*η* ^2^) with interpretations: negligible (*η* ^2^ < 0.01), small (0.01 ≤ *η* ^2^ < 0.06), medium (0.06 ≤ *η* ^2^ < 0.14), and large (*η* ^2^ ≥ 0.14).

##### 2.2.1.2 Mixed‐Effects Analysis

To differentiate between the age groups, linear mixed‐effects models were fitted using the specification:

$$\text{Power}\;\left(\mathrm{dB}\right)\sim C\;\left(\mathrm{Age}\_\text{Group}\right)+\left(1|\text{Patient}\right)$$

where *C* (Age_Group) represents categorical age group effects with the youngest group (0–5 months) as reference, and (1|Patient) captures random patient‐level intercepts. This approach provides parameter estimates (*β* coefficients) representing mean differences between age groups in dB units, with associated 95% confidence intervals. Effect sizes were calculated as Cohen's *d* using the formula *d* = *β*/*σ*_residual, with interpretations: negligible (|*d*| < 0.2), small (0.2 ≤ |*d*| < 0.5), and medium (0.5 ≤ |*d*| < 0.8), and large (|*d*| ≥ 0.8).

#### 2.2.2 Longitudinal Timepoint Comparisons

Within each age group, perioperative EEG changes were analyzed using separate mixed‐effects models:

$$\text{Power}\;\left(\mathrm{dB}\right)\sim C\;\left(\text{Timepoint}\right)+\left(1|\text{Patient}\right)$$

where *C* (Timepoint) represents categorical timepoint effects with baseline as reference. This approach accounts for within‐patient correlations across the perioperative trajectory while providing age‐specific estimates of EEG changes from baseline to LOC, OP, and ROC phases. Models were fitted separately for each age group to allow for age‐specific response patterns and avoid assumptions of parallel temporal effects across developmental stages.

A total of 176 statistical tests were performed: 20 Kruskal‐Wallis tests (4 timepoints × 5 frequency bands), 80 mixed‐effects age group comparisons (4 timepoints × 5 frequency bands × 4 age contrasts), and 76 mixed‐effects timepoint comparisons (4 age groups × 5 frequency bands × variable timepoint contrasts). The analysis included 147 patients with 144–280 observations per cross‐sectional comparison depending on data availability at each timepoint.

#### 2.2.3 Sample Size

Although a formal a priori sample size calculation was not performed for this observational study, post hoc power analysis confirmed adequate statistical power (> 80%) for all significant findings. The achieved sample size (*N* = 147) exceeded requirements for detecting medium‐to‐large effect sizes (*η* ^2^ ≥ 0.06) and represents a large cohort in pediatric EEG anesthesia literature (see Table S5).

## 3 Results

We included 147 children in total, with six aged 0–5 months, 18 aged 6–11 months, 29 aged 12–23 months and 94 ≥ 24 months. Patient characteristics are presented in Table 1. In the infant age group (0–5 months), a higher American Society of Anesthesiologists (ASA) score was observed than in the older group, where the ASA status was evenly distributed. The duration of anesthesia was longer for 6–11‐month‐old children because mostly cleft lip or cleft palate surgeries, which usually have long operating times, were performed. Premedication with p.o. midazolam was not prescribed for children < 6 months of age according to our hospital's standard operating procedures. From 6 months, almost all the children received premedication with midazolam at a similar dosage ranging from 0.59 mg/kg body weight (> 24 months) to 0.77 mg/kg body weight (12–23 months). Inhalational induction was used for children under 6 months. In older children, induction changed from inhalation with sevoflurane to intravenous induction with propofol. From 24 months onwards, children mainly received i.v. induction. For maintenance of anesthesia, mainly sevoflurane was used for up to 23 months. Afterward, in children > 24 months, both sevoflurane (52.9%) and total intravenous anesthesia (TIVA) (44.7%) were used for maintenance of anesthesia (see Table 1).

**TABLE 1:** Baseline characteristics of patients receiving perioperative frontal EEGs.

	0–5 months (*n* = 6)	6–11 months (*n* = 18)	12–23 months (*n* = 29)	> 24 months (*n* = 94)
*Age*				
Months, median (IQR)	3 (2–4)	9 (8–9)	16 (15–20)	67 (63–74)
*Weight*				
Mean kg (SD)	5.3 (0.5)	8.9 (0.3)	10.5 (0.4)	20.1 (0.7)
*ASA*				
ASA I (%)	33.3%	70.6%	69%	77.6%
ASA II (%)	50%	29.4%	31%	18.8%
ASA III (%)	16.7%			3.5%
*Anesthesia duration*				
Duration (mean min, SD)	152 (34)	**222 (25)**	156 (23)	80 (8)
*Premedication*				
Midazolam received	**0%**	94.4%	100%	97.6%
Dosage (mean mg/kg bw + SD)		0.69 (0.06)	0.77 (0.01)	0.59 (0.02)
*Induction of anesthesia*				
Inhalative with sevoflurane	100%	66.7%	61.5%	24.1%
i.v. with propofol		33.3%	38.5%	**75.9%**
*Maintenance of anesthesia*				
Sevoflurane	**100%**	94.1%	89.3%	**52.9%**
Sevoflurane (mean % [SD])	2.17 (0.18)	2.32 (0.07)	2.36 (0.17)	2.29 (0.06)
TIVA		5.9%	7.1%	**44.7%**
TIVA mg/kg/h (mean + SD)		8	7.7 (1.7)	8.8 (0.3)
Mixed			3.6%	2.4%
*Classification of surgery*				
Eye surgery			1 (3.8%)	14 (17.1%)
Cleft lip/cleft palate	1 (16.7%)	10 (66.7%)	11 (42.3%)	2 (2.4%)
ENT‐surgery			1 (3.8%)	16 (19.5%)
Tumor surgery		1 (6.7%)		1 (1.2%)
Smaller operations < 1.5 h	2 (33.3%)	2 (13.3%)	11 (42.3%)	**47 (57.3%)**
Larger operations > 4 h	2 (33.3%)	2 (13.3%)	2 (7.7%)	2 (2.4%)
Bronchoscopy	1 (16.7%)			

*Note:* Data are presented as % or mean ± SD. Bold highlights the most significant values.

Abbreviations: IQR, interquartile range; SD, standard deviation.

### 3.1 Baseline EEG

**FIGURE 2:**
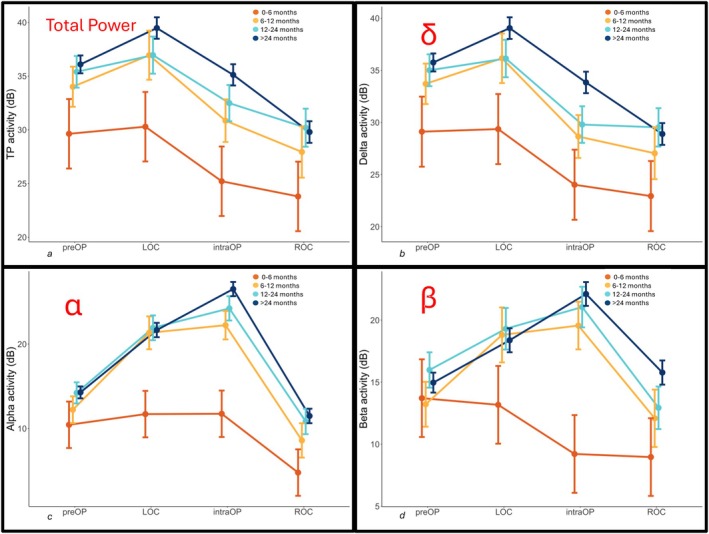
EEG characteristics for each age group. (a) Total power, (b) delta‐power, (c) alpha‐power, and (d) beta‐power. Delta activity was the dominant rhythm at all 4 time points. After the induction of anesthesia, faster frequencies can be observed in children > 6 months. In infants < 6 months, delta activity characterizes the EEG, and faster frequencies (alpha and beta) are not observed. IntraOP, maintenance of anesthesia; LOC, loss of consciousness; PreOP, preoperative; ROC, return of consciousness.

Significant age‐related differences characterized the baseline awake EEG across multiple frequency bands. Delta‐activity dominated all age groups and shows higher values in older children ranging from 24.5 ± 11.5 dB in infants to 32.6 ± 10.3 dB in children ≥ 24 months (*p* = 0.017). Alpha activity demonstrated moderate age‐related increases (*p* = 0.047), while beta power remained similar across groups (*p* = 0.328) (Figure 2) (for detailed comparison see Table 2 and Appendix S1).

**TABLE 2:** Anesthesia concentrations.

	0–5 months (*n* = 6)	6–11 months (*n* = 18)	12–23 months (*n* = 29)	> 24 months (*n* = 94)
*Baseline*				
Midazolam received	**0%**	94.4%	100%	97.6%
Dosage (mean mg/kg bw + SD)		0.69 (0.06)	0.77 (0.01)	0.59 (0.02)
*Loss of consciousness—induction*				
Inhalative with sevoflurane	100%	66.7%	61.5%	24.1%
Sevoflurane (Vol %) (SD)	4.57 (0.85)	4.91 (0.53)	4.25 (0.37)	3.68 (0.27)
i.v. with propofol	0%	33.3%	38.5%	**75.9%**
Propofol mg/kg/h	6.49 (0.56)	5.00 (0.75)	4.68 (0.52)	4.73 (0.20)
Remifentanil	0.20 (0.00)	0.13 (0.01)	0.13 (0.01)	0.16 (0.01)
*OP*				
Sevoflurane	**100%**	94.1%	89.3%	**52.9%**
Sevoflurane (end exp. %) (SD)	2.17 (0.18)	2.32 (0.07)	2.36 (0.17)	2.29 (0.06)
TIVA		5.9%	7.1%	**44.7%**
TIVA mg/kg/h (mean + SD)		8	7.7 (1.7)	8.8 (0.3)
Mixed			3.6%	2.4%
Remifentanil	0.18 (0.02)	0.17 (0.02)	0.14 (0.01)	0.18 (0.01)
*Regain of consciousness*				
Sevo (end exp. %) (SD)		1.02 (0.42)	1.69 (0.32)	0.97 (0.16)

### 3.2 Loss of Consciousness

Anesthetic induction produced dramatic, age‐dependent EEG transformations across all frequency bands (all *p* < 0.05). Alpha oscillations emerged as the most age‐sensitive marker (*p* = 0.021, *η* ^2^ = 0.069), with infants showing minimal modulation while children ≥ 6 months demonstrated substantial power increases (2–3 fold elevation). Beta power followed identical patterns (*p* = 0.008, *η* ^2^ = 0.090). Importantly, infants maintained minimal fast‐frequency activity during LOC, while older children showed robust alpha–beta activation characteristic of GABAergic anesthetic action (Figure 2 and Appendix S1).

### 3.3 Intraoperative EEG

During surgical anesthesia, delta‐power showed the largest effect size (*p* < 0.001, *η* ^2^ = 0.242), with complex age‐dependent patterns. Alpha and beta activities were virtually absent in infants but showed high values in older children (*p* < 0.001, *η* ^2^ = 0.151 and 0.144, respectively). The most significant differences were between 0–5 and 6–11 months, with alpha power showing 1.7‐fold higher values and beta power 2.3‐fold higher values. These developmental EEG differences occurred despite comparable anesthetic concentrations across age groups (sevoflurane: 2.16%–2.47% end‐tidal), indicating that the observed patterns reflect neurophysiological maturation rather than dosing differences. This 6‐month developmental threshold emerged as the critical transition point for the occurrence of higher frequency oscillations, with post hoc analyses showing significant differences between infants and all older groups (*p* < 0.005) but minimal differences among children ≥ 6 months (see Appendix S1).

### 3.4 Return of Consciousness

With the return of consciousness, EEG patterns began converging toward baseline with diminished age‐group differences. Theta power maintained age‐related variation (*p* = 0.014), while alpha activity remained elevated above baseline in children ≥ 6 months. Beta and delta differences became non‐significant, indicating gradual return to age‐appropriate baseline patterns.

## 4 Discussion

In our analysis of perioperative EEG neuromonitoring data, we observed predominant delta‐activity across all age groups from the preoperative awake state until the patients regained consciousness. Following induction of anesthesia, delta‐activity increased from baseline through loss of consciousness and remained elevated during the maintenance phase. At the end of general anesthesia, delta‐activity decreased and returned to baseline levels as the patient regained consciousness.

Children older than 6 months exhibited a marked age‐related increase in alpha‐ and beta‐activity at the time of loss of consciousness, followed by a further increase during intraoperative maintenance. These faster frequencies subsequently declined to baseline levels upon the return of consciousness. In contrast, this perioperative dynamic of alpha‐ and beta‐activity was not observed in children younger than 6 months.

Using full‐montage EEG, Cornelissen et al. extensively studied children under general anesthesia. In a 2015 study, they reported that delta‐activity is present in awake children aged 0–6 months and represents the dominant rhythm. Furthermore, the absolute delta‐power remains relatively constant when comparing preoperative to intraoperative values. Faster frequencies (theta‐ and alpha‐activity) begin to emerge at approximately 4 months of age during anesthesia maintenance [4]. Our findings align with these results, confirming that delta‐activity remains the dominant frequency both preoperatively and intraoperatively. Faster frequencies were minimal in children younger than 6 months. Since our youngest subgroup includes infants up to 5 months of age—an age at which faster frequencies are just beginning to emerge—the separation of frequency bands is less pronounced.

In a separate study conducted at our institution involving newborns and infants aged 0–12 months, alpha‐ and beta‐band activity was first observed at 4 months of age [7]. These findings support the hypothesis that GABAergic synaptogenesis in children under 6 months of age is still developing and therefore does not yet support the generation of characteristic alpha‐oscillations [4].

Beekoo et al. [8] analyzed EEG patterns in patients ranging in age from 1 month to 80 years under general anesthesia with 1 MAC of sevoflurane. In this deeply anesthetized state, no fast frequencies were observed in any age group. This aligns with our findings for children under 6 months, where intraoperative EEG recordings were dominated by delta activity. However, in children older than 6 months, faster frequencies were observed, and with increasing age, alpha power increased significantly. These findings suggest that alpha activity may serve as a useful marker of adequate anesthetic depth in more mature pediatric patients [8].

In a randomized controlled trial by Long et al. [9], 200 children aged 1–6 years received intraoperative sevoflurane titration guided either by EEG neuromonitoring or by standard care. EEG guidance was based on maintaining slow delta‐oscillations in the unprocessed raw EEG as a marker of appropriate anesthetic depth. This approach led to a significant reduction in the sevoflurane concentration—by 88% during induction and 15% during maintenance [9]. Our data show that delta‐oscillations are indeed the dominant frequency in the preoperative state across all age groups. Although delta‐power increases during loss of consciousness, the intraoperative values are comparable to the baseline values. This highlights a challenge: if delta‐power is already high in the awake state, it may be difficult to use it reliably as an indicator of anesthetic depth.

A prospective randomized trial by Sullivan et al. at the Children's Hospital in Boston evaluated whether neuromonitoring via the bispectral index (BIS) in children aged 2–12 years can improve guidance and thereby reduce the sevoflurane concentration intraoperatively. They concluded that the BIS index does not reduce the amount of sevoflurane used intraoperatively and that they do not consider the BIS monitor as a “useful monitor” in the pediatric population [10]. These results are consistent with the results of Tokuwaka et al. [11], in which the BIS value did not fall below the index value of 50 even if the dosage was titrated up to 4.8% sevoflurane; thus, a measurement of the intraoperative anesthesia depth in children between 1 and 2 years of age does not appear valid.

In an observational study from Lee et al., 97 children aged 0–21 years were studied, and raw EEG oscillations were analyzed. Interestingly, power was not observed in the very specific alpha‐range (8–13 Hz) and instead showed a broader span with power at faster frequencies ranging from 12 to 25 Hz [12].

In an observational study by Cornelissen et al., 95 raw EEGs from children aged 0–3 years were recorded during the emergence of anesthesia. Alpha‐activity was present in all children older than 3 months at an end‐expiratory sevoflurane concentration of 2% during surgery. With decreasing sevoflurane concentration, the alpha‐activity decreased until it disappeared. In almost all patients, body movement occurs within 5 min after the loss of alpha‐oscillations [13]. This finding is in line with our results, which revealed prominent alpha‐activity during the maintenance of anesthesia and only minimal values at the emergence of anesthesia for children older than 6 months.

Infants with initially low alpha‐band EEG activity are more likely to develop burst suppression patterns during the maintenance phase of anesthesia [14]. In neonates undergoing cardiac surgery, the presence of burst suppression has been inversely associated with communication outcomes at 5 years of age, and those with prolonged burst suppression episodes—lasting over 90 min—demonstrated the poorest behavioral outcomes postoperatively [15]. Despite these findings, there are currently no established guidelines specifying how to reliably measure anesthetic depth or how to optimize it in neonates and infants. However, certain institutions, such as the Department of Women's and Children's Hospital in Singapore, have implemented specialized training for anesthesiologists in interpreting intraoperative EEG patterns in pediatric patients. This targeted education aims to reduce the risk of both over‐ and undersedation, thereby minimizing the potential for anesthesia‐related neurotoxicity in this vulnerable population [16].

### 4.1 Limitations

The administration of anesthetics was not controlled by a uniform drug protocol, which allows different dosages for each individual patient. The anesthesiologist in charge adhered to the standard operating procedures of our clinic; however, a controlled drug protocol with fixed dosages would ease the comparison between age groups. Unfortunately, ethical reasons make it difficult to perform those studies in these young age groups. End‐tidal sevoflurane concentrations were recorded, but exact effect site concentrations would have been more accurate to compare the age groups. Furthermore, the administration of midazolam was not equal across all groups on the basis of our clinical standard operating procedure, especially with no administration in young children aged 0–5 months. However, a prior study by our group revealed that premedication with midazolam increases the intraoperative alpha‐power in adult patients [17]. ROC was operationalized as first eye opening or observed movement, which may not represent intentional, consciousness‐linked responsiveness; eye opening and purposeful motor responses can be separated by several minutes, and some movements may reflect spinal or subcortical reflexes rather than conscious behavior. Further study limitations include the unbalanced age group design, with the youngest cohort (0–5 months, *n* = 6) having limited statistical power for subgroup analyses. However, the overall sample achieved adequate power for the primary research objectives, and mixed‐effects modeling appropriately handled the inhomogeneous group sizes.

An alternative single mixed‐effects model with age group, timepoint, and interaction terms as fixed effects (plus patient random effects) could holistically test longitudinal age‐time dynamics but was not pursued, as our exploratory focus prioritized reporting of age‐specific EEG power values and pairwise comparisons at each epoch to facilitate future expert interpretation. This modeling choice may limit detection of complex interactions.

EEG analyses relied on proprietary Narcotrend software output without access to raw EEG traces, precluding independent confirmation of artifact sources or EMG contamination. Elevated preoperative delta power may reflect eye‐movement artifacts, potentially confounding age‐related interpretations.

## 5 Conclusion

In this study, we aimed to characterize perioperative EEG dynamics in children aged 1 month to 8 years to better understand age‐specific EEG signatures and ultimately reduce the risk of anesthetic over‐ or underdosing in this vulnerable population. Our findings demonstrate fundamental differences from adult EEG dynamics: notably, delta‐activity consistently remains the dominant rhythm across all perioperative phases in children, including during wakefulness—a pattern distinctly different from that in adults. Furthermore, intraoperative activation of faster frequency bands, such as the alpha and beta bands, is strongly age dependent, becoming evident only from approximately 6 months of age onwards. These developmental differences underscore the importance of age‐adjusted EEG monitoring during pediatric anesthesia.

## Author Contributions

Design of the study: C.S., S.K., and M.M. Contribution to the materials/tools: C.S. and S.K. Data collection: F.P. and S.K. data analysis: F.P., M.M., and S.K. Writing manuscript: M.M., F.P., and S.K. All authors read and approved the final manuscript.

## Funding

The authors have nothing to report.

## Ethics Statement

In this prospective clinical observational study, electroencephalogram (EEG) data were recorded from children with approval from the Charité—University Medicine Berlin ethics committee (EA2/027/15). The study was registered at clinicaltrials.gov under the number: NCT02481999. The data were collected at the Campus Virchow Klinik (CVK) of the Charité—Universitätsmedizin Berlin, which spans from 08.09.2015 to 24.05.2017. EEG data from infants (Clinicaltrials.gov Registration: NCT04093661) were collected from 2017 until 2019 with the same inclusion and exclusion criteria with approval from the Charité—University Medicine Berlin ethics committee (EA2/115/19). Both patient cohorts were combined for further EEG processing and final analysis. The study adhered to the Declaration of Helsinki.

## Consent

The consent for participation of the patients was obtained from their parents.

## Conflicts of Interest

C.S. received grants or contracts and nonfinancial support from the German Research Society, German Aerospace Center, Einstein Foundation Berlin, Federal Joint Committee (G‐BA), Inner University Grants, Project Management Agency, Non‐Profit Society Promoting Science and Education, European Society of Anaesthesiology and Intensive Care, BMWI—Federal Ministry for Economic Affairs and Climate Action, Georg Thieme Verlag, Dr. F. Köhler Chemie GmbH, Sintetica GmbH, Max‐Planck‐Gesellschaft zur Förderung der Wissenschaften e.V., Stifterverband für die deutsche Wissenschaft e.V., Metronic, Philips Electronics Nederland BV, BMBF (Federal Ministry of Education and Research, RKI, The European Commission Horizont Europa, Prothor, Takeda Pharmaceutical Company Limited, Association of the Scientific Medical Societies in Germany, German Research Foundation, German National Academy of Sciences—Leopoldina, Berliner Medizinische Gesellschaft, European Society of Intensive Care Medicine, European Society of Anaesthesiology and Intensive Care, German Society of Anaesthesiology and Intensive Care Medicine, German Interdisciplinary Association for Intensive Care and Emergency Medicine, German Sepsis Foundation and holds various international patents; these holdings have not affected any decisions regarding his research or this study). S.K. was funded by the Deutsche Forschungsgemeinschaft (DFG, German Research Society)—Project number KO 4249/3‐1; she is an inventor on patents sold to Medtronic. She received speakers' fees from Medtronic, and personal fees from Georg Thieme Verlag and Springer Verlag. All remaining authors declare that they have no conflicts of interest.

## Supporting information


**Appendix S1:** pan70156‐sup‐0001‐AppendixS1.docx.

## Data Availability

The data that support the findings of this study are available from the corresponding author upon reasonable request.
