# Supplementary material for: EEG Dynamics in Children Before, During and After General Anesthesia
Source: Paediatr Anaesth. 2026 Mar 5;36(6):641–9. doi: 10.1002/pan.70156 (PMC13161511; doi:10.1002/pan.70156)
Supplement: Supplementary file 1 — Appendix S1: pan70156‐sup‐0001‐AppendixS1.docx. [file PAN-36-641-s001.docx]

**Title: EEG dynamics in children before, during and after general anesthesia**

Running Title: EEG Signatures Across Pediatric Anesthesia

Maximilian Markus.^1^, Feidias Panagiotou ^1^, Claudia Spies^1^, Susanne Koch ^1^

^1^Department of Anaesthesiology and Intensive Care Medicine (CCM, CVK), Charité – Universitätsmedizin Berlin, corporate member of Freie Universität Berlin and Humboldt-Universität zu Berlin

**Correspondence to:**

Dr. med. Maximilian Markus

Maximilian.markus@charite.de
Klinik für Anästhesiologie und Intensivmedizin
Charité - Universitätsmedizin Berlin
Charitéplatz 1
10117 Berlin

Germany

Content

[I. Frequency Analysis 3](#_Toc223516859)

[II. Cross Sectional Analysis 5](#_Toc223516860)

[III. Post Hoc Analysis 6](#_Toc223516861)

[IV. Mixed Effect Model Differences between Age Groups at Timepoints 8](#_Toc223516862)

[V. Sample Size calculation 11](#_Toc223516863)

# Frequency Analysis

| Suppl Table 1 | | Detailed Analysis of the frequency band analysis at each time point | | | | |
| --- | --- | --- | --- | --- | --- | --- |
|  |  | |  |  |  |  |
|  | 0–5 Months | | 6–11 Months | 12–23 Months | >24 Months | P Values |
| Baseline | | | | | | |
| Total Power | 1312  (409; 2215) | | 3442  (2102; 4782) | 4557  (3308; 5805) | 5286  (4330; 6243) | 0,002 |
| Beta | 49,95  (-30; 130) | | 31  (12; 49) | 59  (33; 85) | 44  (36; 52) | 0,177 |
| Alpha | 16  (1; 30) | | 23  (14; 31) | 31  (23; 40) | 31  (27; 35) | 0,034 |
| Theta | 70  (21; 118) | | 150  (98; 201) | 229  (147; 311) | 232  (194; 269) | 0,001 |
| Delta | 11815  (347; 2016) | | 3238  (1954; 4522) | 4236  (3042; 5430) | 4978  (4039; 5917) | 0,002 |
| Loss of Consciousness | | | | | | |
| Totale Power | 4406  (-3968; 12782) | | 6832  (2806; 10858) | 6392  (4134; 8651) | 11111  (9357; 12864) | 0,001 |
| Beta | 31  (-1; 65) | | 103  (67; 139) | 106  (74; 138) | 95  (71; 118) | 0,019 |
| Alpha | 30  (-5; 67) | | 187  (108; 265) | 194  (139; 250) | 216  (151; 281) | 0,004 |
| Theta | 109  (9; 208) | | 418  (254; 582) | 338  (216; 460) | 331  (257; 406) | 0,019 |
| Delta | 4239  (-4134; 12614) | | 6123  (2183; 10062) | 5752  (3490; 8014) | 10467  (8711; 12223) | 0,001 |
| Intraoperative | | | | | | |
| Totale Power | 501  (199; 802) | | 1404  (969; 1839) | 2194  (1601; 2786) | 4230  (3560; 4900) | 0,001 |
| Beta | 19  (-5; 44) | | 113  (77; 149) | 148  (113; 182) | 196  (166; 225) | 0,001 |
| Alpha | 27  (6; 48) | | 228  (142; 315) | 310  (223; 397) | 534  (453; 615) | 0,001 |
| Theta | 44  (18; 71) | | 188  (113; 263) | 253  (180; 325) | 400  (303; 497) | 0,001 |
| Delta | 410  (121; 698) | | 873  (587; 1159) | 1482  (922; 2041) | 3141  (2541; 3741) | 0,001 |
| Return of Consciousness | | | | | | |
| Totale Power | 348  (-18; 715) | | 1168  (-147; 2484) | 2175  (1105; 3245) | 1993  (1184; 2802) | 0,039 |
| Beta | 57  (-74; 189) | | 31  (1; 61) | 28  (17; 40) | 64  (44; 84) | 0,004 |
| Alpha | 7  (-6; 20) | | 12  (1; 23) | 20  (6; 33) | 18  (14; 22) | 0,005 |
| Theta | 11  (4; 19) | | 36  (7; 65) | 75  (43; 107) | 58  (32; 84) | 0,002 |
| Delta | 271  (23; 520) | | 1087  (-207; 2382) | 2051  (1006; 3096) | 1851  (1060; 2642) | 0,07 |

Data presented as mean µV² + (95% CI)

# Cross Sectional Analysis

Suppl. Table 2 summarizes the results of Kruskal–Wallis tests comparing absolute EEG power across four pediatric age groups (1–6 months, 6–11 months, 12–23 months, ≥24 months) at each perioperative epoch. Significant age-related differences were most pronounced in theta power at baseline, intraoperative maintenance, and emergence, and in delta power during maintenance (all p < 0.05), with large effect sizes during maintenance (η² ≥ 0.14). Alpha and total power showed smaller but measurable age effects at baseline and induction. Post-hoc pairwise comparisons identified specific group contrasts driving these effects.

| Suppl Table 2 Differences between age groups (Kruskal Wallis Test) | | | | | | |
| --- | --- | --- | --- | --- | --- | --- |
| Frequency Band | H Statistic | df | p-value | η² (Eta-squared) | ε² (Epsilon-squared) | Effect Size |
| Baseline | | | | | | |
| TP | 9.849 | 3 | 0.020 | 0.049 | 0.000475 | Small |
| Beta | 3.442 | 3 | 0.328 | 0.003 | 0.000166 | Negligible |
| Alpha | 7.936 | 3 | 0.047 | 0.035 | 0.000383 | Small |
| Theta | 18.485 | 3 | <0.001 | 0.111 | 0.000891 | Medium |
| Delta | 10.197 | 3 | 0.017 | 0.051 | 0.000492 | Small |
| Loss of consciousness | | | | | | |
| TP | 12.013 | 3 | 0.007 | 0.092 | 0.001155 | Medium |
| Beta | 11.844 | 3 | 0.008 | 0.090 | 0.001139 | Medium |
| Alpha | 9.766 | 3 | 0.021 | 0.069 | 0.000939 | Medium |
| Theta | 8.583 | 3 | 0.035 | 0.057 | 0.000825 | Small |
| Delta | 8.951 | 3 | 0.030 | 0.061 | 0.000860 | Medium |
| Intraoperative | | | | | | |
| TP | 22.325 | 3 | <0.001 | 0.186 | 0.001914 | Large |
| Beta | 18.007 | 3 | <0.001 | 0.144 | 0.001544 | Large |
| Alpha | 18.713 | 3 | <0.001 | 0.151 | 0.001604 | Large |
| Theta | 13.816 | 3 | 0.003 | 0.104 | 0.001185 | Medium |
| Delta | 28.186 | 3 | <0.001 | 0.242 | 0.002417 | Large |
| Return of consciousness | | | | | | |
| TP | 6.976 | 3 | 0.073 | 0.042 | 0.000712 | Small |
| Beta | 2.242 | 3 | 0.524 | 0.000 | 0.000229 | Negligible |
| Alpha | 8.287 | 3 | 0.040 | 0.056 | 0.000846 | Small |
| Theta | 10.617 | 3 | 0.014 | 0.080 | 0.001083 | Medium |
| Delta | 5.675 | 3 | 0.129 | 0.028 | 0.000579 | Small |

This table presents H statistics, degrees of freedom (df), p-values, eta-squared (η²) and epsilon-squared (ε²) effect sizes, significance levels, total sample sizes, and the number of significant pairwise age-group comparisons for each frequency band (delta, theta, alpha, beta, total power) at four perioperative timepoints (Baseline, Loss of Consciousness, Maintenance, Return of Consciousness) TP=Total Power.

# Post Hoc Analysis

Supplementary analyses using Bonferroni-corrected Mann–Whitney U tests identified specific age-group contrasts driving overall Kruskal–Wallis effects. At baseline, total power (TP) differed between the youngest (1–6 months) and oldest (≥24 months) groups (p=0.007, r=0.276), and theta power (Theta) showed large differences between 1–6 months versus 12–23 months and versus ≥24 months (both p≤0.003, r≥0.305), as well as between 6–11 months and ≥24 months (p=0.002, r=0.303). During induction, beta power (Beta) differed between 1–6 months and older groups (p≤0.008, r≥0.322), and alpha power (Alpha) was lower in 1–6 months versus each other group (p≤0.005, r≥0.335). Intraoperative maintenance revealed widespread contrasts, with the youngest group consistently different from all older age groups across TP, Beta, Alpha, Theta, and Delta (all p≤0.003, r≥0.354). At return of consciousness, only theta power remained significantly elevated in older infants compared to the youngest group (p=0.006, r=0.544).

| Suppl Table 3 Post Hoc Analysis | | | | | | | |
| --- | --- | --- | --- | --- | --- | --- | --- |
| Timepoint | Frequency Band | Overall p-value | Comparison | N1 | N2 | Post-hoc p-value | Effect Size (r) |
| Base | TP | 0.020 | Age 1 vs Age 4 | 6 | 91 | 0.007 | 0.276 |
| Base | Theta | <0.001 | Age 1 vs Age 3 | 6 | 29 | 0.002 | 0.526 |
| Base | Theta | <0.001 | Age 1 vs Age 4 | 6 | 91 | 0.003 | 0.305 |
| Base | Theta | <0.001 | Age 2 vs Age 4 | 18 | 91 | 0.002 | 0.303 |
| Base | Delta | 0.017 | Age 1 vs Age 4 | 6 | 91 | 0.004 | 0.294 |
| LOC | Beta | 0.008 | Age 1 vs Age 3 | 6 | 21 | 0.002 | 0.610 |
| LOC | Beta | 0.008 | Age 1 vs Age 4 | 6 | 63 | 0.008 | 0.322 |
| LOC | Alpha | 0.021 | Age 1 vs Age 2 | 6 | 12 | 0.002 | 0.727 |
| LOC | Alpha | 0.021 | Age 1 vs Age 3 | 6 | 21 | 0.004 | 0.550 |
| LOC | Alpha | 0.021 | Age 1 vs Age 4 | 6 | 63 | 0.005 | 0.335 |
| OP | TP | <0.001 | Age 1 vs Age 2 | 6 | 16 | 0.002 | 0.668 |
| OP | TP | <0.001 | Age 1 vs Age 3 | 6 | 22 | <0.001 | 0.660 |
| OP | TP | <0.001 | Age 1 vs Age 4 | 6 | 64 | 0.002 | 0.370 |
| OP | TP | <0.001 | Age 2 vs Age 4 | 16 | 64 | 0.002 | 0.354 |
| OP | Beta | <0.001 | Age 1 vs Age 2 | 6 | 16 | 0.002 | 0.647 |
| OP | Beta | <0.001 | Age 1 vs Age 3 | 6 | 22 | 0.003 | 0.568 |
| OP | Beta | <0.001 | Age 1 vs Age 4 | 6 | 64 | <0.001 | 0.407 |
| OP | Alpha | <0.001 | Age 1 vs Age 2 | 6 | 16 | <0.001 | 0.767 |
| OP | Alpha | <0.001 | Age 1 vs Age 3 | 6 | 22 | <0.001 | 0.756 |
| OP | Alpha | <0.001 | Age 1 vs Age 4 | 6 | 64 | 0.002 | 0.375 |
| OP | Theta | 0.003 | Age 1 vs Age 2 | 6 | 16 | 0.002 | 0.647 |
| OP | Theta | 0.003 | Age 1 vs Age 3 | 6 | 22 | 0.004 | 0.541 |
| OP | Theta | 0.003 | Age 1 vs Age 4 | 6 | 64 | 0.001 | 0.385 |
| OP | Delta | <0.001 | Age 1 vs Age 4 | 6 | 64 | 0.001 | 0.387 |
| OP | Delta | <0.001 | Age 2 vs Age 4 | 16 | 64 | <0.001 | 0.454 |
| OP | Delta | <0.001 | Age 3 vs Age 4 | 22 | 64 | 0.002 | 0.332 |
| ROC | Theta | 0.014 | Age 1 vs Age 3 | 6 | 20 | 0.006 | 0.544 |

This table lists pairwise Mann–Whitney U test results for significant age-group differences in absolute EEG power for each frequency band and perioperative epoch. For each comparison, N1 and N2 denote sample sizes, and p-values and effect sizes (r) reflect Bonferroni-corrected significance. Age groups are coded as 1 = 1–6 months, 2 = 6–11 months, 3 = 12–23 months, and 4 = ≥ 24 months. TP=Total Power

# Mixed Effect Model Differences between Age Groups at Timepoints

Suppl. Table 4 reports mixed-effects model estimates of perioperative EEG power changes within each age group. For each frequency band, the model compares power at Loss of Consciousness, Intraoperative Maintenance, and Return of Consciousness against Baseline (reference), adjusting for repeated measures. Children ≥24 months exhibited significant increases in alpha and beta power during induction and maintenance (β > 5950 dB, p < 0.001, large d), followed by a marked decrease at emergence (β ≈ –3425 dB, p = 0.001). Children 6–11 months showed robust timepoint effects in alpha, beta, and theta bands (p < 0.01, large d).

| Supple Table 4 | | | | | | |
| --- | --- | --- | --- | --- | --- | --- |
| Frequency  Band | Age Group Comparison | β (dB) | p-value | 95% CI | Cohen's d | Effect Size |
| Baseline | | | | | | |
| Alpha | Age 4 vs Ref | 3128.130 | <0.001 | [1613.075, 4643.185] | 0.989 | Large |
| Alpha | Age 2 vs Ref | 1302.056 | 0.298 | [-1150.092, 3754.203] | 0.412 | Small |
| Alpha | Age 3 vs Ref | 439.075 | 0.664 | [-1540.915, 2419.064] | 0.139 | Negligible |
| Alpha | Age Group Var vs Ref | 1.000 | nan | [nan, nan] | 0.000 | Negligible |
| Beta | Age 4 vs Ref | -1171.557 | 0.092 | [-2535.599, 192.485] | -0.278 | Small |
| Beta | Age 3 vs Ref | 935.609 | 0.427 | [-1374.752, 3245.970] | 0.222 | Small |
| Beta | Age 2 vs Ref | -1143.778 | 0.524 | [-4664.514, 2376.958] | -0.272 | Small |
| Beta | Age Group Var vs Ref | 1.000 | nan | [nan, nan] | 0.000 | Negligible |
| Delta | Age 4 vs Ref | 8171.980 | <0.001 | [5198.114, 11145.846] | 1.135 | Large |
| Delta | Age 3 vs Ref | 7397.443 | 0.008 | [1910.004, 12884.881] | 1.027 | Large |
| Delta | Age 2 vs Ref | 7547.000 | 0.012 | [1641.966, 13452.034] | 1.048 | Large |
| Delta | Age Group Var vs Ref | 1.000 | nan | [nan, nan] | 0.000 | Negligible |
| TP | Age 4 vs Ref | 3267.905 | 0.329 | [-3290.200, 9826.010] | 0.475 | Small |
| TP | Age 3 vs Ref | 3304.713 | 0.344 | [-3533.386, 10142.811] | 0.480 | Small |
| TP | Age 2 vs Ref | 2459.056 | 0.561 | [-5826.977, 10745.089] | 0.357 | Small |
| TP | Age Group Var vs Ref | 1.000 | nan | [nan, nan] | 0.000 | Negligible |
| Theta | Age 4 vs Ref | 9106.092 | <0.001 | [5284.445, 12927.739] | 1.706 | Large |
| Theta | Age 3 vs Ref | 8881.161 | <0.001 | [3633.131, 14129.190] | 1.664 | Large |
| Theta | Age 2 vs Ref | 2436.444 | 0.318 | [-2341.177, 7214.066] | 0.456 | Small |
| Theta | Age Group Var vs Ref | 1.000 | nan | [nan, nan] | 0.000 | Negligible |
| Loss of consciousness | | | | | | |
| Alpha | Age 2 vs Ref | 9625.833 | 0.005 | [2916.301, 16335.366] | 1.988 | Large |
| Alpha | Age 4 vs Ref | 7813.722 | 0.008 | [2080.472, 13546.972] | 1.614 | Large |
| Alpha | Age 3 vs Ref | 8372.643 | 0.008 | [2160.823, 14584.463] | 1.729 | Large |
| Alpha | Age Group Var vs Ref | 1.000 | nan | [nan, nan] | 0.000 | Negligible |
| Beta | Age 3 vs Ref | 9815.643 | <0.001 | [4268.843, 15362.442] | 2.271 | Large |
| Beta | Age 4 vs Ref | 7509.833 | 0.004 | [2390.369, 12629.297] | 1.737 | Large |
| Beta | Age 2 vs Ref | 8591.250 | 0.005 | [2600.022, 14582.478] | 1.987 | Large |
| Beta | Age Group Var vs Ref | 1.000 | nan | [nan, nan] | 0.000 | Negligible |
| Delta | Age 4 vs Ref | 9856.548 | 0.035 | [681.714, 19031.381] | 1.272 | Large |
| Delta | Age 3 vs Ref | 9399.833 | 0.064 | [-540.848, 19340.515] | 1.213 | Large |
| Delta | Age 2 vs Ref | 8360.250 | 0.127 | [-2376.913, 19097.413] | 1.079 | Large |
| Delta | Age Group Var vs Ref | 1.000 | nan | [nan, nan] | 0.000 | Negligible |
| TP | Age 4 vs Ref | 12088.960 | 0.015 | [2361.761, 21816.160] | 1.472 | Large |
| TP | Age 3 vs Ref | 10616.405 | 0.048 | [77.250, 21155.560] | 1.293 | Large |
| TP | Age 2 vs Ref | 10371.167 | 0.074 | [-1012.422, 21754.755] | 1.263 | Large |
| TP | Age Group Var vs Ref | 1.000 | nan | [nan, nan] | 0.000 | Negligible |
| Theta | Age 4 vs Ref | 7733.698 | 0.012 | [1692.921, 13774.476] | 1.516 | Large |
| Theta | Age 2 vs Ref | 8688.500 | 0.016 | [1619.073, 15757.927] | 1.703 | Large |
| Theta | Age 3 vs Ref | 7894.571 | 0.018 | [1349.554, 14439.589] | 1.548 | Large |
| Theta | Age Group Var vs Ref | 1.000 | nan | [nan, nan] | 0.000 | Negligible |
| Intraoperative | | | | | | |
| Alpha | Age 4 vs Ref | 12794.438 | <0.001 | [9240.933, 16347.942] | 2.261 | Large |
| Alpha | Age 2 vs Ref | 12298.875 | <0.001 | [7383.923, 17213.827] | 2.174 | Large |
| Alpha | Age 3 vs Ref | 13218.409 | <0.001 | [7290.754, 19146.064] | 2.336 | Large |
| Alpha | Age Group Var vs Ref | 1.000 | nan | [nan, nan] | 0.000 | Negligible |
| Beta | Age 4 vs Ref | 13102.979 | <0.001 | [10193.278, 16012.680] | 2.828 | Large |
| Beta | Age 2 vs Ref | 11247.042 | <0.001 | [7222.552, 15271.531] | 2.428 | Large |
| Beta | Age 3 vs Ref | 11043.212 | nan | [nan, nan] | 2.384 | Large |
| Beta | Age Group Var vs Ref | 1.000 | nan | [nan, nan] | 0.000 | Negligible |
| Delta | Age 4 vs Ref | 6753.859 | <0.001 | [4341.949, 9165.769] | 1.015 | Large |
| Delta | Age 3 vs Ref | 3135.045 | 0.055 | [-66.593, 6336.684] | 0.471 | Small |
| Delta | Age 2 vs Ref | -338.375 | 0.887 | [-5002.134, 4325.384] | -0.051 | Negligible |
| Delta | Age Group Var vs Ref | 1.000 | nan | [nan, nan] | 0.000 | Negligible |
| TP | Age 4 vs Ref | 11375.734 | <0.001 | [8788.865, 13962.603] | 1.568 | Large |
| TP | Age 3 vs Ref | 13605.227 | <0.001 | [8718.890, 18491.565] | 1.875 | Large |
| TP | Age 2 vs Ref | 11972.063 | <0.001 | [6907.641, 17036.484] | 1.650 | Large |
| TP | Age Group Var vs Ref | 1.000 | nan | [nan, nan] | 0.000 | Negligible |
| Theta | Age 4 vs Ref | 9324.219 | <0.001 | [7425.125, 11223.313] | 1.818 | Large |
| Theta | Age 3 vs Ref | 8473.273 | <0.001 | [6131.255, 10815.290] | 1.652 | Large |
| Theta | Age 2 vs Ref | 7937.375 | <0.001 | [4320.338, 11554.412] | 1.547 | Large |
| Theta | Age Group Var vs Ref | 1.000 | nan | [nan, nan] | 0.000 | Negligible |
| Return of consciousness | | | | | | |
| Alpha | Age 4 vs Ref | 6113.161 | <0.001 | [5186.765, 7039.558] | 1.817 | Large |
| Alpha | Age 3 vs Ref | 4718.400 | <0.001 | [2022.091, 7414.709] | 1.402 | Large |
| Alpha | Age 2 vs Ref | 3867.545 | 0.015 | [763.684, 6971.407] | 1.150 | Large |
| Alpha | Age Group Var vs Ref | 1.000 | nan | [nan, nan] | 0.000 | Negligible |
| Beta | Age 4 vs Ref | 3929.371 | 0.159 | [-1538.574, 9397.316] | 0.803 | Large |
| Beta | Age 3 vs Ref | 4056.000 | 0.176 | [-1820.596, 9932.596] | 0.829 | Large |
| Beta | Age 2 vs Ref | 3716.955 | 0.267 | [-2847.216, 10281.125] | 0.760 | Medium |
| Beta | Age Group Var vs Ref | 1.000 | nan | [nan, nan] | 0.000 | Negligible |
| Delta | Age 4 vs Ref | 5235.720 | <0.001 | [2524.405, 7947.036] | 1.018 | Large |
| Delta | Age 3 vs Ref | 4925.683 | <0.001 | [2110.648, 7740.718] | 0.958 | Large |
| Delta | Age 2 vs Ref | 4106.152 | 0.102 | [-813.548, 9025.851] | 0.798 | Medium |
| Delta | Age Group Var vs Ref | 1.000 | nan | [nan, nan] | 0.000 | Negligible |
| TP | Age 3 vs Ref | 8812.050 | <0.001 | [6200.178, 11423.922] | 1.334 | Large |
| TP | Age 4 vs Ref | 7091.355 | 0.006 | [2042.637, 12140.073] | 1.074 | Large |
| TP | Age 2 vs Ref | 6307.955 | 0.040 | [283.690, 12332.219] | 0.955 | Large |
| TP | Age Group Var vs Ref | 1.000 | nan | [nan, nan] | 0.000 | Negligible |
| Theta | Age 3 vs Ref | 7041.033 | 0.002 | [2662.958, 11419.109] | 1.566 | Large |
| Theta | Age 4 vs Ref | 4975.930 | 0.008 | [1325.304, 8626.556] | 1.107 | Large |
| Theta | Age 2 vs Ref | 3888.061 | 0.043 | [129.395, 7646.726] | 0.865 | Large |
| Theta | Age Group Var vs Ref | 1.000 | nan | [nan, nan] | 0.000 | Negligible |

Mixed-effects model results for each age cohort (1–6 months, 6–11 months, 12–23 months, ≥24 months) showing β coefficients (dB), 95% confidence intervals, Cohen’s d, and p-values for EEG band power differences at Loss of Consciousness, Maintenance, and Return of Consciousness relative to Baseline

# Sample Size calculation

The present study was conceived as an exploratory investigation of pediatric electroencephalographic dynamics during general anesthesia, drawing on naturally occurring surgical cases rather than a predetermined hypothesis framework. Consequently, no a priori sample size calculation was performed. Instead, a comprehensive post-hoc power analysis was undertaken to verify that the cohort of 147 participants offered sufficient sensitivity to detect the effects observed across four perioperative timepoints. At the baseline assessment (N = 144), small effect sizes in total power (η² = 0.049), alpha absolute power (η² = 0.035), and delta absolute power (η² = 0.051), as well as a medium effect in theta absolute power (η² = 0.111), all achieved statistical power exceeding 80%. During loss of consciousness (N = 102), medium effects in total and beta absolute power (η² = 0.092 and 0.090, respectively), alpha absolute power (η² = 0.069), and delta absolute power (η² = 0.061), alongside a small effect in theta absolute power (η² = 0.057), were similarly well powered. Intraoperative analyses (N = 108) revealed large effects in delta (η² = 0.242), total power (η² = 0.186), alpha (η² = 0.151), and beta absolute power (η² = 0.144), plus a medium effect in theta absolute power (η² = 0.104), all surpassing the 80% threshold. Finally, return-of-consciousness assessments (N = 99) demonstrated adequate power for medium effects in theta (η² = 0.080) and small effects in alpha absolute power (η² = 0.056). These findings confirm that, despite its exploratory design, the sample size achieved robust statistical sensitivity for all significant EEG band differences, thereby supporting the validity of the study’s conclusions.

| Suppl. Table 5 Sample Size calculation | | | | | | | |
| --- | --- | --- | --- | --- | --- | --- | --- |
| Frequency  Band | H_Statistic | P_Value | Eta_Squared | Effect_Size  Category | Sample  Size N | Statistical  Power | Power  Adequacy |
| Baseline | | | | | | | |
| TP | 9.849 | 0.020 | 0.049 | Small | 144 | ≥80% | Adequate |
| Alpha | 7.936 | 0.047 | 0.035 | Small | 144 | ≥80% | Adequate |
| Theta | 18.485 | <0.001 | 0.111 | Medium | 144 | ≥80% | Adequate |
| Delta | 10.197 | 0.017 | 0.051 | Small | 144 | ≥80% | Adequate |
| Loss of consciousness | | | | | | | |
| TP | 12.013 | 0.007 | 0.092 | Medium | 102 | ≥80% | Adequate |
| Beta | 11.844 | 0.008 | 0.09 | Medium | 102 | ≥80% | Adequate |
| Alpha | 9.766 | 0.021 | 0.069 | Medium | 102 | ≥80% | Adequate |
| Theta | 8.583 | 0.035 | 0.057 | Small | 102 | ≥80% | Adequate |
| Delta | 8.951 | 0.030 | 0.061 | Medium | 102 | ≥80% | Adequate |
| intraoperative | | | | | | | |
| TP | 22.325 | <0.001 | 0.186 | Large | 108 | ≥80% | Adequate |
| Beta | 18.007 | <0.001 | 0.144 | Large | 108 | ≥80% | Adequate |
| Alpha | 18.713 | <0.001 | 0.151 | Large | 108 | ≥80% | Adequate |
| Theta | 13.816 | 0.003 | 0.104 | Medium | 108 | ≥80% | Adequate |
| Delta | 28.186 | <0.001 | 0.242 | Large | 108 | ≥80% | Adequate |
| Return of consciousness | | | | | | | |
| Alpha | 8.287 | 0.040 | 0.056 | Small | 99 | ≥80% | Adequate |
| Theta | 10.617 | 0.014 | 0.08 | Medium | 99 | ≥80% | Adequate |
